# Hippocampal sclerosis is associated with celiac disease type immunity in patients with drug-resistant temporal lobe epilepsy

**DOI:** 10.1007/s00415-024-12210-w

**Published:** 2024-02-10

**Authors:** Maria Peltola, Katri Kaukinen, Pabitra Basnyat, Jani Raitanen, Katri Haimila, Suvi Liimatainen, Sirpa Rainesalo, Jukka Peltola

**Affiliations:** 1 Department of Psychiatry, Tampere University Hospital, Tampere, Finland; 2 Coeliac Disease Research Centre, Faculty of Medicine and Health Technology, Tampere University, 33014 Tampere, Finland; 3 Department of Internal Medicine, Tampere University Hospital, Tampere, Finland; 4 Department of Neurology, Faculty of Medicine and Health Technology, Tampere University, Tampere, Finland; 5 Faculty of Social Sciences, Health Sciences, Tampere University, Tampere, Finland; 6 Immunogenetics Laboratory, Finnish Red Cross Blood Service, Vantaa, Finland; 7 Department of Neurology, Tampere University Hospital, Tampere, Finland; 8 Administration Centre, Tampere University Hospital, Pirkanmaa Wellbeing County, Tampere, Finland; 9 Division of Acute Medicine, Tampere University Hospital, Tampere, Finland; 10 UKK Institute for Health Promotion Research, Tampere, Finland; 11 Vanha Vaasa Hospital, Vierinkiventie 1, 65380 Vaasa, Finland

**Keywords:** Anti-gliadin antibody, Celiac disease, Gluten sensitivity, Hippocampal sclerosis, Refractory epilepsy, Systemic immunity

## Abstract

**Background:**

A prior small-scale single center study suggested an association between celiac disease (CD)-type immunity and refractory temporal lobe epilepsy (TLE) with hippocampal sclerosis (HS). The present study addresses this putative association in a large, well-characterized group of drug-resistant epilepsy (DRE) patients. These patients were grouped based on the spectrum of CD and gluten sensitivity-associated antibodies.

**Methods:**

In this cross-sectional study, 253 consecutive adult epilepsy patients (135 females, 118 males; age 16–76 years) were categorized into three groups: (i) CD-positive group with either prior diagnosis of CD or CD-specific TG2/EmA antibodies, (ii) AGA-positive group with antigliadin antibodies (AGA) but without CD, and (iii) CD/AGA-negative group without any gluten sensitivity-associated antibodies or CD. Clinical and immunological findings were then compared among the groups.

**Results:**

TLE with HS was more common in the CD-positive group compared to CD/AGA-negative group (31.8% versus 11.9%, *P* = 0.019). Autoimmune disorders were more common in the AGA-positive group than in the CD/AGA-negative group (*P* = 0.025). Considering HS lateralization; left lateralization was more common in CD-positive group compared to CD/AGA-negative group (71.4% versus 25%, *P* = 0.030). TG6 seropositivity did not differ among the groups (*P* > 0.05).

**Conclusions:**

This study provides further evidence linking TLE with HS and CD-type autoimmunity suggesting that CD-type immune response to gluten can be one potential mechanism as a disease modifier leading to DRE and HS. Understanding these immunological factors is imperative for developing immunomodulatory or dietary treatments for DRE potentially preventing HS progression.

## Introduction

Epilepsy is not a single disease entity but rather a group of disorders reflecting underlying brain dysfunction that may result from many different causes. Drug-resistant epilepsy (DRE) is defined as failure to achieve sustained seizure freedom by adequate trials of two tolerated and appropriately chosen and used anti-seizure medication (ASM) regimens [1]. Temporal lobe epilepsy (TLE) is the most common adult focal epilepsy type, often associated with hippocampal sclerosis (HS). In TLE patients with HS, resistance to ASMs is common, making HS as one of the most prevailing pathologies of DRE [2]. Transient insults like prolonged febrile seizures, brain trauma, or status epilepticus can induce epileptogenesis, resulting in HS with refractory seizures [3]. TLE with HS is considered a potentially progressive disorder leading to increased neuronal damage over time. MRI studies have shown lesions not only in the hippocampal area but also more extensively in the brain [4].

Lately, there has been increasing interest in the role of the gut in controlling autoimmune neuroinflammation [5]. In celiac disease (CD), it has been reported that 67% of newly diagnosed patients exhibit neurological manifestations [6]. Moreover, accumulating data also supported the association between multiple systemic autoimmune disorders including CD and increased risk of epilepsy [7]. CD is a chronic autoimmune-mediated condition driven by ingestion of the dietary wheat gluten component, gliadin. It is characterized by a variable combination of gluten-related signs and symptoms, and disease-specific transglutaminase 2 (TG2) and endomysial antibodies (EmA) in addition to enteropathy. Genetic susceptibility, particularly human leukocyte antigen (HLA)-DQ2 and/or HLA-DQ8 haplotypes, is essential for CD development [8]. Currently, a life-long gluten-free diet is the only treatment for CD.

Recently, it has been recognized in some cases that gluten-containing cereals may induce wide range of symptoms even in the absence of CD. Non-celiac gluten sensitivity is now considered a distinct entity from CD, as patients with it lack enteropathy, EmA or TG2 antibodies, or HLA types.

However, anti-gliadin antibodies (AGA) are often positive in this group [9]. Interestingly, both CD and non-celiac gluten sensitivity have been associated with neurologic and psychiatric diseases, such as epilepsy, ataxia, cerebellar degeneration, neuropathy, depression, psychosis, schizophrenia, bipolar disorder, and autistic spectrum disorders [6, 10, 11].

In addition to gut, transglutaminase enzyme isomers, such as TG2 and TG6, are abundantly expressed in brain and dysfunction of TG2 activity is closely associated with many neurodegenerative diseases, such as Alzheimer’s disease and Parkinson’s disease [12, 13]. Furthermore, the other isoform TG6, expressed in the brain is considered the autoantigen in gluten-related disorders (GRD) with primary neurological symptoms [5, 14], and 40% of newly diagnosed CD patients with neurological manifestations have antibodies to TG6 [6]. Moreover, in gluten ataxia, a link to anti-glutamic acid decarboxylase (GAD) antibodies has been suggested [15], which is noteworthy as anti-GAD antibodies have been frequently reported in patients with TLE [16–18].

The prevalence of TG6 antibodies has not been thoroughly investigated in drug-resistant patients with GRD cases or specifically in TLE with HS and GRD patients. A small Italian study reported that 17% of patients with epilepsy and posterior cerebral calcifications had anti-TG6 antibodies, compared with none of the patients with focal epilepsy of unknown etiology [19]. Another case identified high levels of IgA class TG6 in a patient with CD, epilepsy, and cerebral calcifications [20].

Previously in our pilot study, we found a significant link between CD type gluten sensitivity and drug-resistant TLE with HS, highlighting the need for a detailed clinical characterization of epilepsy [21]. To our knowledge, subsequent studies have not addressed this association between HS and celiac immunity or investigated anti-TG6 antibody prevalence in this specific group of patients. Therefore, the goal of the present study was to analyze CD and gluten sensitivity-associated antibodies in a large, well-characterized group of patients with drug-resistant focal epilepsy by categorizing epilepsy patients into three different groups: (i) those with either prior diagnosis of CD or CD-specific TG2/EmA antibodies (CD-positive group), (ii) those with AGA but without CD (AGA-positive group), and (iii) those without any gluten sensitivity-associated antibodies or CD (CD/AGA-negative group). We also explored whether lateralization of HS was associated with gluten immunity, considering the differential immune functions of the left and right cerebral hemispheres [22].

## Materials and methods

### Study patients

We conducted a cross-sectional study comprising 253 consecutive adult drug-resistant patients with epilepsy in the Outpatient Clinic of Neurology at Tampere University Hospital. The Ethics Committee of Tampere University Hospital approved the study protocol, and all the patients provided written informed consent according to the Declaration of Helsinki. Epilepsy was classified according to the International League Against Epilepsy (ILAE) guidelines [23]. Focal epilepsy types were categorized into TLE, frontal lobe (FLE), parietal lobe (PLE), occipital lobe (OLE), multifocal, or unknown focal epilepsies. If the patient had TLE, the patient was further designated to either the TLE with HS group or the TLE without HS group, depending on the presence of HS.

The definition of the epilepsy type was based on patient history, electroclinical findings (EEG/video EEG), neuroimaging results, and etiology. Most patients had undergone standard brain MRI (1.5 Tesla) with a specific epilepsy protocol evaluated by a neuroradiologist. Data on the duration of epilepsy, epilepsy surgery, and all previous and current ASM were collected. Patients with refractory epilepsy had been evaluated for the possibility of epilepsy surgery. Patients with dementia, moderate, or severe mental retardation or malignant brain tumors with epilepsy were excluded from the study. Data regarding concomitant autoimmune diseases were collected. The presence or absence of CD was not part of the inclusion criteria because the study was specifically designed to evaluate the CD- or AGA-specific antibodies alterations in the epilepsy patient group. The presence or absence of ataxia was assessed based on medical records.

## Study design

### Study groups

The drug-resistant patients with epilepsy were classified into three groups:

(i) **CD-positive group:** consisted of patients with positive CD-specific antibodies meaning immunoglobulin A (IgA) and immunoglobulin G (IgG) class EmA and TG2 antibodies. In addition, the group includes patients who were previously diagnosed with biopsy-proven CD (they were treated with gluten-free diet and thus without detectable antibodies in the present study).
(ii) **AGA-positive group:** consisted of patients with IgA and/or IgG AGA without serological evidence of CD-specific EmA or TG2-antibodies, and no previous diagnosis of CD. In the current study, this group is recognized as non-celiac gluten sensitivity.
(iii) **CD/AGA-negative group:** epilepsy patients without current or previously diagnosed CD and without AGA.

**Fig. 1:**
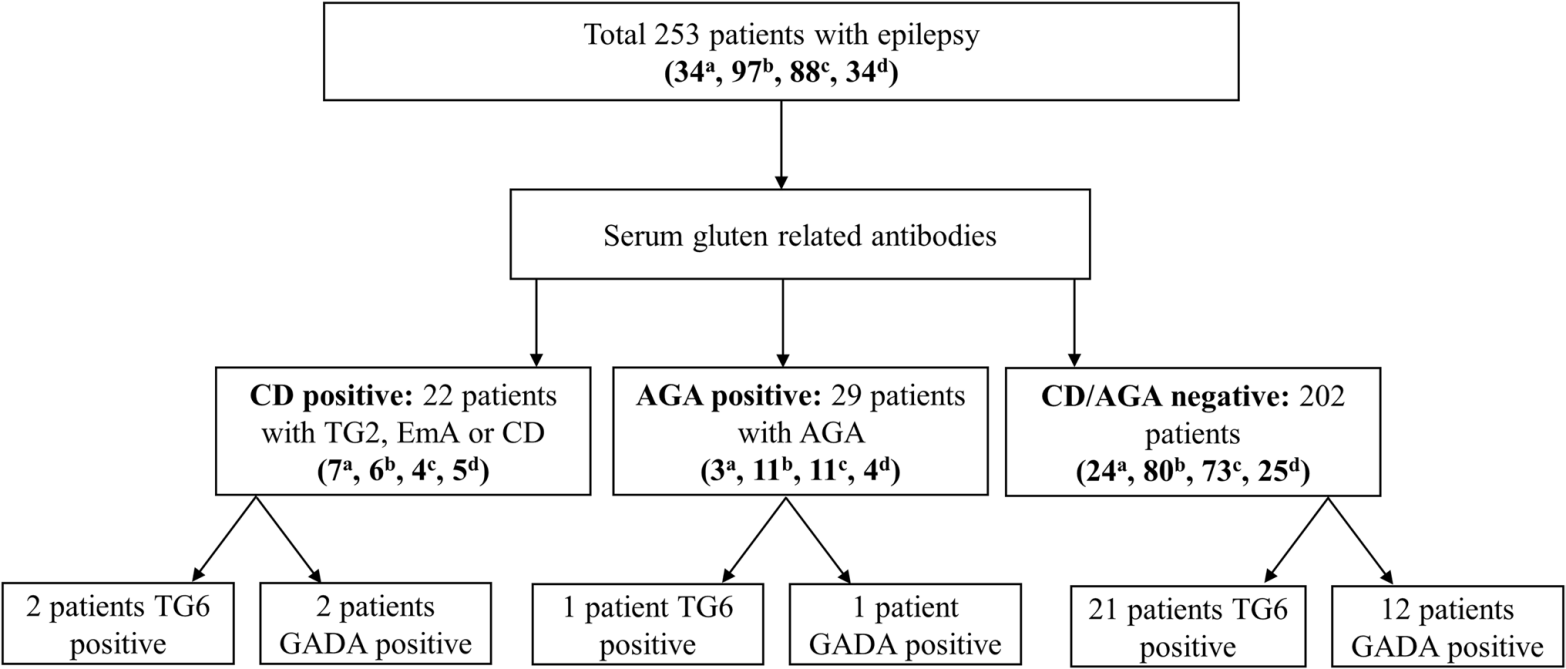
Flowchart of patient enrollment. CD-positive group consisted of patients with (TG2) antibodies and endomysial (EmA) antibodies including the patients previously diagnosed with biopsy-proven CD. AGA-positive group consisted of patients with (AGA) antibodies without serological evidence of CD-specific TG2 or EmA antibodies. CD/AGA-negative group consisted of patients without serological evidence of any gluten-related antibodies or without previously diagnosed CD. ^a^Temporal lobe epilepsy with hippocampal sclerosis. ^b^Temporal lobe epilepsy without hippocampal sclerosis. ^c^Other focal epilepsies. ^d^Generalized epilepsies (one juvenile absence epilepsy, nine idiopathic generalized epilepsy, and 24 juvenile myoclonic epilepsy). *AGA* Anti-gliadin antibody, *CD* Celiac disease, *EmA* Endomysial antibody, *GADA* Glutamic acid decarboxylase antibody, *TG2* Tissue transglutaminase 2 antibody, *TG6* Tissue transglutaminase 6 antibody

In addition, we presented the distribution of TG6 antibodies and glutamic acid decarboxylase (GAD) antibodies in different groups. All these study groups are categorized in the flowchart which includes the frequencies of positive antibodies in the study patients and their respective distribution according to their epilepsy types (Fig. 1).

### Immunological analyses

Blood samples were collected during scheduled outpatient visits at Tampere University Hospital. Serum IgA and IgG-class AGA were measured according to standard enzyme immunoassay with gliadin as antigen (Sigma G3375). The lower limit of positivity for IgA class AGA was 0.2 EU/ml and for IgG-class 10 EU/ml. Serum IgA- and IgG-class TG2 antibodies were measured by enzyme-linked immunosorbent assay (ELISA) using a commercial kit (Celikey, Phadia, GmbH, Freiburg, Germany) according to the manufacturer`s instructions. Values TG2 IgA > 3.0 U/l and TG2 IgG > 10 U/l were considered positive. In TG2-antibody positive subjects, IgA- and IgG-class EmA were determined by an indirect immunofluorescence method using human umbilical cord as substrate [24], and a dilution of 1: > 5 was regarded positive.

Serum IgA class antibodies against TG6 were detected by ELISA using human recombinant TG6 (0312GE00-FWD, Zedira, Darmstadt, Germany) as antigens according to the manufacturer’s instructions. Concentrations over 41 U/ml were considered positive for TG6 antibodies.

Glutamic acid decarboxylase antibody (GADA) was measured by radioimmunoassay (RIA) at the Hospital for Children and Adolescents, University of Helsinki, Finland and at the Service of Neurology and the Institut d ´Investigacions Biomèdiques August Pi i Sunyer (IDIBAPS) as previously described [16].

### Statistical analysis

The normality of distribution was tested using the Shapiro–Wilk test and examining kurtosis and skewness values. Descriptive statistics (frequencies and proportions or means and standard deviations (SD)) were reported to summarize patients’ characteristics (Table 1). Due to the positive skewed distribution of continuous variables, Mann–Whitney *U* test was applied to analyze differences between groups. When examining an association between groups and categorized variables, Pearson’s chi-squared test, if assumptions were valid, or Fisher’s exact test was used. Because two tests were used for each background characteristics and measurements, we used Bonferroni correction i.e., *P* < 0.025 was considered significant. All analyses were conducted using Stata statistical software version 16.1 (StataCorp, College Station, Texas, USA).

**Table 1:** Demographics and clinical characteristics of the patients with reference to epilepsy types

Characteristics	TLE with HS	TLE without HS	XTLE	IGE
*n*	34	97	88	34
F/M	21/13	45/52	47/41	22/12
Age, years, *mean (SD)*	45 (13.3)	40 (14.1)	37 (14.2)	30 (13.5)
Age at onset, years, *mean (SD)*	14.5 (10.7)	18.5 (13.1)	17 (15.6)	13 (3.6)
Etiology of epilepsy, *n (%)*				
*Structural*	34 (100.0)	43 (44.3)	49 (55.7)	–
*Genetic*	–	–	–	34 (100.0)
*Infectious*	–	8 (8.2)	2 (2.3)	–
*Immune*		3 (3.1)	1 (1.1)	
*Unknown*	–	43 (44.3)	36 (40.9)	–
Duration of epilepsy, years, *mean (SD)*	31 (15.9)	22.2 (14.4)	21 (14.8)	18 (15.0)
Autoimmune disease including CD,* n (%)*	5 (14.7)	9 (9.3)	6 (6.8)	2 (5.9)
Autoimmune disease without CD, *n (%)*	–	6 (6.2)	4 (4.5)	1 (2.9)
HS lateralization (right/left/bilateral)	20/13/1	–	–	–

Data is shown as mean (SD) or n (%), as appropriate

HS, hippocampal sclerosis; TLE, temporal lobe epilepsy; XTLE, extra-temporal lobe epilepsy; IGE, idiopathic generalized epilepsy

## Results

### Clinical characteristics of epilepsy

This study included a total of 253 patients with epilepsy, of whom 34 had TLE with HS, 97 had TLE without HS, 88 had other focal epilepsies, and 34 had generalized epilepsies. Clinical characteristics of patients with different epilepsy types are presented in Table 1.

### Clinical and laboratory finding in three study groups: celiac disease type immunity, anti-gliadin immunity and patients without any of the above

Altogether 22 patients were CD-positive, 29 patients were AGA-positive and the remaining 202 patients were antibody-negative. Out of 22 CD-positive patients, 5 patients (23%) were positive for AGA. TLE with HS was more common in the CD-positive group than in CD/AGA-negative group (31.8% versus 11.9%, *P* = 0.019). CD-positive group had longer duration of epilepsy (28.0 versus 21.6 mean years, *P* = 0.032), and there were more females in this group compared to the CD/AGA-negative group (72.7% versus 51.0%, *P* = 0.052). Autoimmune disorders excluding CD were more common in the AGA-positive group compared to the CD/AGA-negative group (13.8% versus 3.0%, *P* = 0.025).

HS lateralization was different between CD-positive and CD/AGA groups (*P* = 0.030): left lateralization was more common in CD-positive groups (71.4%) compared with CD/AGA-negative epilepsy group (25%) (Table 2).

**Table 2:** Clinical characteristics and laboratory findings in three study groups: celiac-type autoimmunity, anti-gliadin immunity and patients without any of the above

Characteristics	CD-positive	AGA-positive	CD/AGA-negative	*P* value^1^	*P* value^2^
*n*	22	29	202		
Female, *n *(%)	16 (72.7)	16 (55.2)	103 (51.0)	*0.052*^*a*^	0.67^a^
Age, years, mean (SD)	41.3 (13.0)	36.9 (14.9)	38.9 (14.7)	0.46^b^	0.41^b^
Age at onset, years, mean (SD)	13.2 (9.6)	13.3 (11.2)	17.4 (13.6)	0.23^b^	0.12^b^
Duration of epilepsy, years, mean (SD)	28.0 (13.6)	23.4 (14.3)	21.6 (15.3)	*0.032*^*b*^	0.42^b^
Etiology, *n *(%)				0.36^a^	**0.004**^**a**^
*Structural*	9 (40.9)	11 (37.9)	106 (52.5)		
*Infectious*	2 (9.1)	1 (3.4)	7 (3.5)		
*Immune*	0	3 (10.3)	1 (0.5)		
*Genetic*	5 (22.7)	4 (13.8)	25 (12.4)		
*Unknown*	6 (27.3)	10 (34.5)	63 (31.2)		
Epilepsy type, *n *(%)				**0.019**^**a**^	0.98^a^
*TLE with HS*	7 (31.8)	3 (10.3)	24 (11.9)		
*TLE without HS*	6 (27.3)	11 (37.9)	80 (39.6)		
*XTLE*	4 (18.2)	11 (37.9)	73 (36.1)		
*IGE*	5 (22.7)	4 (13.8)	25 (12.4)		
HS with dual pathology, *n *(%)	3 (42.9)	0	4 (16.7)	0.14^a^	0.44^a^
HS lateralization (right/left/bilateral), *n *(%)	1/5/1 (14.3/71.4/14.3)	1/2/0 (33.3/66.7/0.0)	18/6/0 (75/25/0)	*0.030*^*c*^	0.20^c^
Seizure-free patients for 1 year before labs, *n *(%)	4 (18.2)	2 (6.9)	40 (19.8)	0.85^c^	0.06^c^
At least one seizure 1 month before labs, *n *(%)	13 (59.1)	20 (69.0)	124 (61.4)	0.83^a^	0.43^a^
Seizure frequency 1 month before labs, *n *(%)	1.0 (0–13)	2.0 (0–60)	1.0 (0–95)	0.68^b^	0.35^b^
Autoimmune disease*, *n *(%)	1 (4.5)	4 (13.8)	6 (3.0)	0.52^c^	**0.025**^**c**^
TG6 seropositivity, *n *(%)	2 (10.0)	1 (3.4)	21 (11.4)	1.00^c^	0.32^c^
GAD autoantibodies, *n *(%)	2 (9.1)	1 (3.4)	12 (5.9)	0.63^c^	0.71^c^

Data is shown as mean (SD) or n (%), as appropriate

*CD-positive group* consisted of patients with (TG2) antibodies and endomysial (EmA) antibodies including the patients previously diagnosed with biopsy-proven CD

*AGA-positive group* consisted of patients with (AGA) antibodies without serological evidence of CD-specific TG2 or EmA antibodies

*CD/AGA-negative group* consisted of patients without serological evidence of any gluten-related antibodies or without previously diagnosed CD

^1^Comparison between CD-positive and the CD/AGA-negative group; ^2^Comparison between AGA-positive and the CD/AGA-negative group

*P* < 0.025 significant with Bonferroni correction-bolded, borderline significant *P* = 0.025–0.050 with italics.

^a^Pearson’s chi-squared test; ^b^Mann–Whitney *U* test; ^c^Fisher’s exact test;

*excluding CD

There was no significant difference in the TG6 seropositivity among the CD-positive group, AGA-positive group, and CD/AGA-negative group (*P* > 0.05, Table 2). When we compared the prevalence of TG6 in the CD-positive/AGA-positive combined group and the CD/AGA-negative group, the results showed no significant difference in the prevalence of TG6 antibodies; 3 (6.1%) CD-positive/AGA-positive patients were seropositive, and 21 (11.4%) CD/AGA-negative were seropositive for TG6 (*p* = 0.279).

Among the epilepsy types, the prevalence of TG6 antibodies was 4 of 29 (13.8%) in TLE with HS, 7 of 92 (7.6%) in TLE without HS, 8 of 81 (9.9%) in XTLE, and 5 of 31 (16.1%) in IGE. The proportion of TG6 seropositivity varied among the epilepsy types, but the difference was not statistically significant (*P* > 0.05). The TG6 results could not be obtained for 20 patients due to sample deficiency.

GAD autoimmunity was not associated with CD or AGA immunity in patients with HS compared to the CD/AGA-negative group (Table 2). There was only 1 GADA-positive TLE with HS patient in CD-positive group, no GADA-positive HS patients in the AGA-positive group, and 2 GADA-positive HS patients in the TG2 negative group.

The proportion of epilepsy patients with immune etiology was higher in AGA-positive group compared to CD/AGA-negative group (10.3% vs. 0.5%, *P* = 0.004), while none of the drug-resistant epilepsy patients in the CD-positive group had evidence of separate immune etiology other than CD.

Seven of 34 HS patients had MRI evidence of dual pathology: vascular malformation (*N* = 3), CNS infection (*N* = 1) and cortical dysplasia (CD) (*N* = 3). Proportionately, TLE with HS patients with dual pathology tended to be more common in CD-positive group compared to CD/AGA-negative group (42.9% vs. 16.8%, *P* > 0.05). Furthermore, none of the HS patients in AGA-positive group had dual pathology (Table 2).

## Discussion

This study provides further evidence of an association between TLE with HS and CD-type autoimmunity suggesting that CD-type immune response to gluten can be one potential mechanism as a disease modifier leading to intractable epilepsy and HS. First, we confirmed the association between HS and CD-type autoimmunity reported in our small-scale pilot study [21] in a large, well-characterized group of patients with DRE. Second, we provided new evidence of the significance of lateralization of HS in patients with CD-type autoimmunity. Finally, contrary to the other gluten-related neurological disorders such as gluten ataxia, no association with anti-TG6 antibodies was found in patients with DRE.

Robust association between CD and TLE with HS was found in the present study. In epilepsy patients with CD, the prevalence of TLE was similar to patients with AGA or without CD/AGA immunity.

However, the proportion of patients with HS was three-fold in patients with CD suggesting a strong association with CD and hippocampal damage. These findings are in line with our previous findings involving 48 DRE patients with epilepsy, where seven patients had signs of CD -type autoimmunity and all had TLE with HS. Duodenal biopsies in the pilot study showed that three of the seven patients had histological evidence of CD, and four had inflammatory changes consistent with early CD without villous atrophy [21]. A recent population-based study demonstrated the frequent co-occurrence of epilepsy and autoimmune diseases. The risk of epilepsy was significantly heightened, especially in children with autoimmune disease (< 18 years), with the highest odds ratios for systemic lupus erythematosus and CD [11]. However, this study did not address the issues regarding DRE or epilepsy types but provided a significant association between CD and epilepsy.

In the present study, 75% of the patients with CD-type immunity had left-sided HS compared to 25% of patients without CD-type immunity, suggesting a possibility for lateralized cerebral influence of immune processes of gluten-associated immunity. Interestingly, in our recent study with the same patient group, IL-6 levels and the IL-6/IL-10 ratio were differentially regulated among patients with TLE depending on the presence of HS and its lateralization [25].

To the best of our knowledge, this is the first large epilepsy cohort in which patients have been tested for antibodies against TG6. Notably, TG6 antibodies showed no associations with other gluten-related antibodies, epilepsy types, or HS. In our epilepsy cohort, 10% (24/253) of patients tested positive for TG6 antibodies, which significantly differs from the frequency of TG6 antibodies reported in other neurological manifestations of GRD [10]. On the other hand, our results are in line with a previous study reporting 14% prevalence of TG6 autoantibodies in a cohort of 86 consecutive Finnish classic CD patients without known neurologic symptoms [26]. Despite TG6 being considered a specific marker for gluten ataxia [26, 27], our findings suggest that TG6 antibodies cannot be considered a diagnostic biomarker for DRE with HS-associated gluten sensitivity. Importantly, none of the DRE patients in our study exhibited ataxia, suggesting distinct mechanisms between gluten ataxia and epilepsy in this specific cohort. The difference between gluten ataxia and epilepsy may also be attributed to the higher expression of TG6 in the deeper brain structures, including the midbrain, brainstem, and possibly cerebellum but not in hippocampus [28]. This makes it likely that TG6 antibodies primarily affect these structures, causing neurological symptoms, such as ataxia and movement disorders, but not epilepsy.

In our study, GAD autoimmunity was not associated with CD or AGA immunity in patients with HS compared to the CD/AGA-negative group. GAD antibodies are one of the most frequently detected antibodies in patients with adult-onset chronic intractable seizures [29] and are also found in patients with focal epilepsy associated with progressive mesial TLE, limbic encephalitis and cerebellar ataxia [30, 31]. Notably, a recent study found a significantly higher frequency of GAD antibodies in CD patients than in controls (12.5% vs. 1.1%) [32].

Pathophysiological mechanisms linking gluten with epilepsy are not fully understood, but several studies have indicated immune-mediated mechanisms, such as gluten-mediated toxicity, immune-induced cortical damage, and malabsorption [33]. Interestingly, an experimental epilepsy study hypothesized that the toxic effects of gliadin peptide on kainate-induced neurotoxicity could be mediated by the involvement of transglutaminases [34]. TG2 is directly involved in neuro-inflammation associated with chronic neurodegenerative disorders including Alzheimer’s disease and Parkinson’s disease and play a pathogenic role in neurodegenerative disease by promoting the aggregation of disease-specific proteins that accumulate in these disorders [13]. A possible role of TG2-mediated NF-κB activation pathway, the main regulator of inflammation, was suggested in the pathogenesis of Alzheimer’s disease, Parkinson’s disease, multiple sclerosis and amyotrophic lateral sclerosis [35]. In addition, NF-κB activation may also be mediated by different cytokines and chemokines associated with and released during the course of CD [35].

Despite scanty evidence, understanding of gluten and the gut–brain axis has provided a basis for exploring the effectiveness of a gluten-free diet in supporting the management of epilepsy in patients with CD. It is suggested that the earlier the gluten-free diet was implemented after the onset of seizures, the better the likelihood of the gluten-free diet being successful in supporting control of seizures [33]. A recent systematic review of gluten sensitivity and epilepsy demonstrated the efficacy of gluten-free diet in 53% of epilepsy patients with gluten sensitivity or CD either by reducing frequency of seizures, enabling reduced doses of anti-seizure medications or even stopping these medications [36]. Multiple studies have reported clinical benefits of gluten-free diet in epilepsy patients with gluten sensitivity, CD and gluten ataxia [6, 37, 38].

This study has some limitations. We did not include a healthy control group for the primary measurements because the focus of this study was to evaluate variability within a group of patients with DRE. The study included cross-sectional cohort with no prespecified MRI or other protocols to address the evolution of HS or other neurodegenerative changes in MRI. We measured only levels of TG6 IgA class autoantibodies in this study and the present cohort lacked duodenal biopsy for all gluten sensitivity patients. A measurement of Ig-G class antibodies also would have provided more information, or we could have found few more cases of gluten sensitivity. However, Hadjivassiliou and co-workers recently reported that all TG6-positive patients (39/100) had Ig-A class antibodies, and 11% of those patients also had Ig-G class antibodies [6]. Similarly, in a previous study, only 12/86 (14%) of CD patients (Finnish patients as in our present study) had TG6 antibodies, and 75% of them had IgA class TG6 antibodies [26]. A similar finding was also reported in patients with dermatitis herpetiformis, while TG6 positivity was found in 13/33 (39%), with IgA detected in 11 patients, IgG in three, and both in one patient [39]. In addition, the prevalence of AGA seropositivity also in healthy subjects presents diagnostic challenges for gluten sensitivity. AGA are common in the general population and in the Finnish population, the prevalence of AGA in the elderly population was 14% and only 10% of them had also TG2 [40].

In conclusion, this study further supports an association between TLE with HS and CD-type autoimmunity suggesting that CD-type autoimmune responses against dietary gluten may potentially be involved and modify disease processes leading to intractable epilepsy and HS. Blood screening for CD in DRE is important. However, unlike gluten ataxia, in patients with drug-resistant epilepsy, TG6 antibodies do not reliably identify gluten-sensitive patients. Increasing our understanding of these immunological factors may contribute to the development of immunomodulatory or dietary treatments for DRE and open the possibility for prevention of HS progression in patients with TLE. Future studies are needed to explore the underlying mechanisms resulting this association and to utilize CD and gluten-associated antibodies as predictors of epilepsy prognosis. Moreover, prospective studies are warranted in patients with DRE and CD or gluten sensitivity to evaluate the therapeutic effect of gluten-free diet, especially in TLE. Our patients had a long duration of epilepsy with established HS, so a gluten-free diet is unlikely to be effective in these patients.

## Data Availability

The data that support the findings of this study are available from the corresponding author upon reasonable request.

## Abbreviations

CD: Celiac Disease
TG6: Transglutaminase 6
AGA: Anti-gliadin antibody
TLE: Temporal lobe epilepsy
HS: Hippocampal sclerosis
